# Surgical management of primary gastric Ewing-like sarcoma at the lesser curvature

**DOI:** 10.1093/jscr/rjad498

**Published:** 2023-09-08

**Authors:** Xinlin Chin, Scott Cooper, Priscilla Martin

**Affiliations:** Department of General Surgery, Sunshine Coast University Hospital, Birtinya, Queensland, Australia; School of Medicine, Griffith University, Birtinya, Queensland, Australia; College of Medicine & Dentistry, James Cook University, Mackay, Queensland, Australia; Department of General Surgery, Sunshine Coast University Hospital, Birtinya, Queensland, Australia; Department of General Surgery, Sunshine Coast University Hospital, Birtinya, Queensland, Australia

**Keywords:** Ewing sarcoma, Ewing-like sarcoma, gastric Ewing sarcoma, Ewing sarcoma of the stomach, Extraskeletal Ewing sarcoma

## Abstract

We report the case of a 65-year-old female who presented with a 6-month history of epigastric pain and dyspepsia. Computed tomography of the abdomen and pelvis showed an enhancing nodular lesion and an indeterminate 4 mm lymph node on the lesser curvature of the stomach raising concerns for gastric malignancy. Upper gastrointestinal endoscopy revealed a 10 cm malignant appearing lesion along the gastric lesser curvature. Histopathology demonstrated spindled and small round blue cell tumor with immunohistochemistry staining consistent with Ewing-like sarcoma. After multidisciplinary team discussion the patient was arranged for neoadjuvant chemotherapy with early re-imaging, followed by consideration of gastrectomy. This case highlights the unusual diagnosis of primary gastric Ewing-like sarcoma and the management of this rare condition.

## Introduction

Ewing sarcoma (ES), first reported by James Ewing in 1921 is a rare and highly malignant small round cell tumor that usually arises in skeletal tissues [1, 2]. It lacks neuroectodermal differentiation and usually occurs in the long axial bones, femur, and pelvis [1, 2]. Extraskeletal ES is an unusual, aggressive soft tissue tumor with high incidence of recurrence and metastasis [1–3]. It was first reported in 1975 and accounts for 15–20% of the ES cases [1, 2]. Extraskeletal ES have been found in the retroperitoneum, paraspinal region, and limbs but ES in the stomach is extremely rare [1]. To date, only 13 cases of gastric ES have been reported in the literature [1]. We present a case of primary gastric Ewing-like Sarcoma, the endoscopic and radiological findings, surgical management, and review of literature.

### Case report

**Figure 1:**
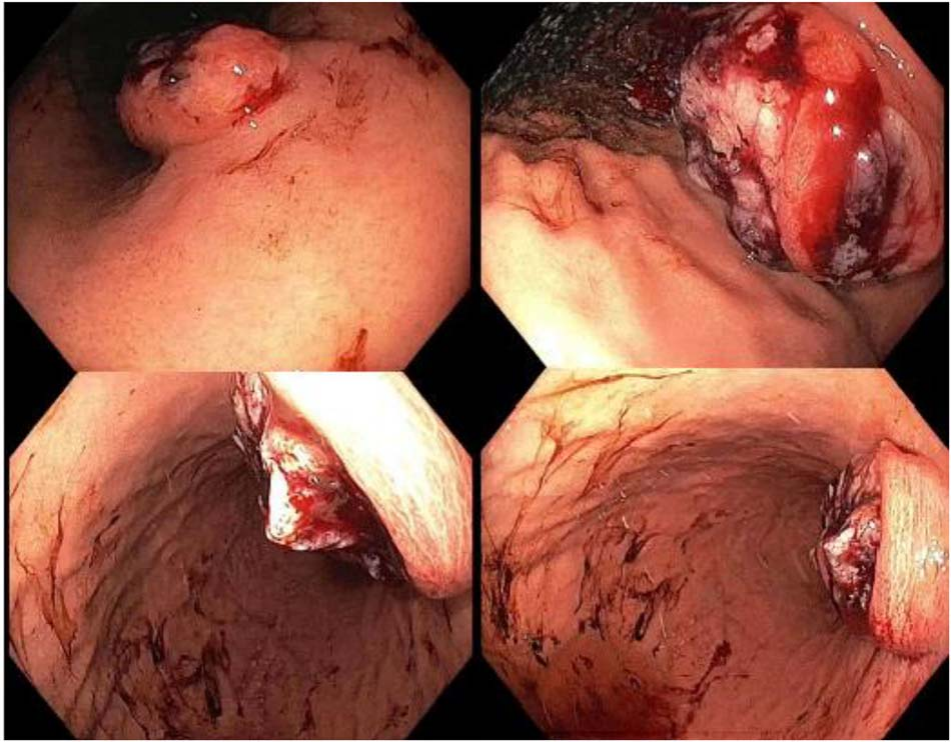
Upper gastrointestinal endoscopy showing a fungating, malignant appearing tumor along the gastric lesser curvature.

**Figure 2:**
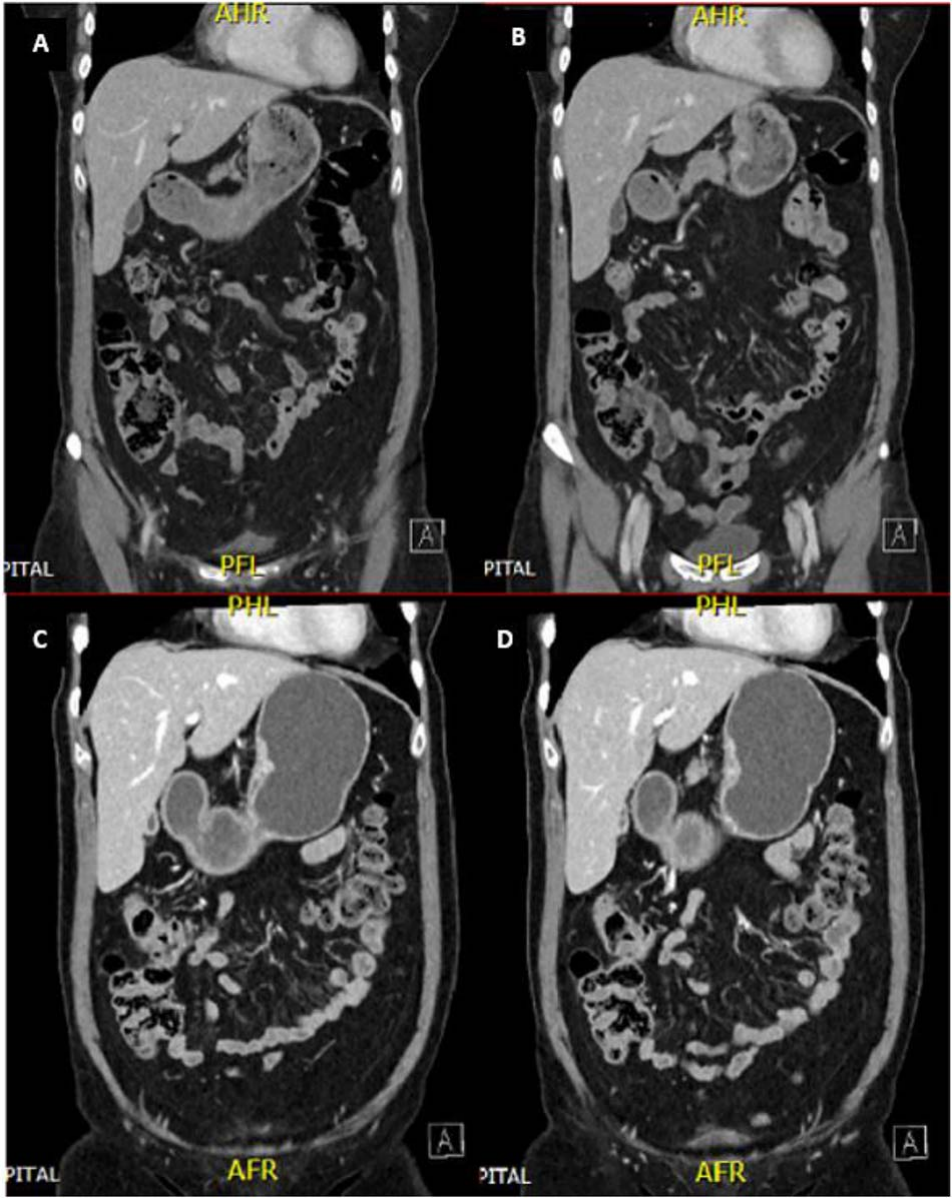
CT abdomen and pelvis demonstrated an enhancing nodular lesion along the gastric lesser curvature (A and B). Interval CT abdomen and pelvis showed reduced gastric wall thickening following 7 weeks of chemotherapy (C and D).

**Figure 3:**
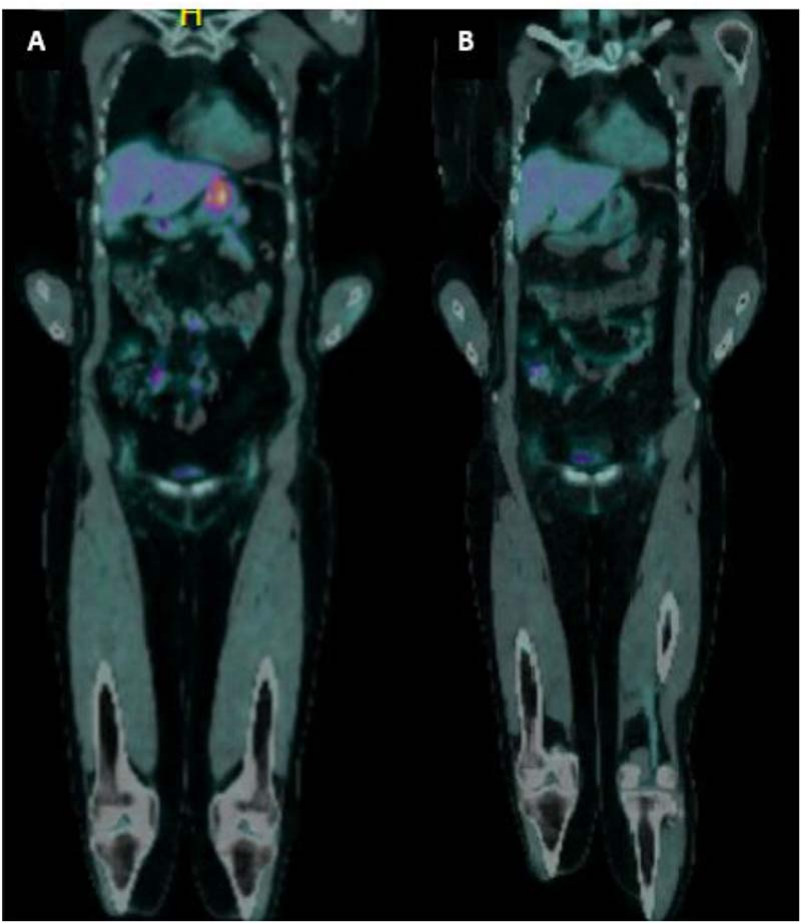
(a) PET imaging demonstrated intense FDG uptake along the lesser curvature of the stomach. (b) Interval PET imaging demonstrated reduced FDG uptake at the gastric lesser curvature and reduced gastric wall thickening.

A 65-year-old female was referred to the general surgical outpatient clinic with a 6-month history of epigastric pain and dyspepsia. The patient’s only other medical history was eczema. Physical examination revealed a soft abdomen with no palpable mass. A 10 cm fungating, malignant appearing mass was identified along the gastric lesser curvature on upper gastrointestinal endoscopy (Fig. 1). Histopathology of the biopsied mass demonstrated spindled and small round blue cell tumor with immunohistochemistry staining consistent with Ewing-like sarcoma. Computed tomography (CT) of the abdomen and pelvis showed an enhancing nodular lesion with central calcification and an indeterminate 4 mm lymph node along the lesser curvature of the stomach which raised concerns for gastric malignancy (Fig. 2). Intense fluorodeoxyglucose (FDG) uptake was noted along the lesser curvature of the stomach on staging positron emission tomography (PET) (Fig. 3). There was no evidence of distant metastasis.

The patient was planned for a 12-week neoadjuvant chemotherapy following discussion with the sarcoma unit and upper gastrointestinal multidisciplinary meeting but her treatment was ceased on the 7th week due to side effects from her chemotherapy regime (Doxorubicin, Vincristine, Cyclophosphamide, Etoposide, Ifosfamide). Interval CT of the abdomen and PET imaging showed reduced FDG uptake at the gastric lesser curvature and reduced gastric wall thickening (Figs 2 and 3).

Staging laparoscopy was unremarkable and the patient proceeded to have an open subtotal proximal gastrectomy. Intraoperatively, there was a 2 cm palpable tumor at the proximal gastric lesser curvature. There was no nodal, hepatic, and peritoneal involvement. Proximal gastrectomy was performed and reconstructed with a handsewn end-to-side oesophago-gastric anastomosis. This technique was decided upon due to this being a sarcoma (as opposed to adenocarcinoma) and therefore an extensive nodal resection was not required.

The resected specimen measured 20 × 20 mm. Histological examination revealed that the tumor developed from the submucosa and muscularis propria layers, extending into the subserosal connective tissue. The tumor cells were characterized by highly atypical small to intermediate round to spindle cells embedded in a fibrotic stroma and mixed with foamy macrophages. No evidence of metastasis was found in the 11 lymph nodes and the surgical margins were clear with 75% tumor regression. Staining was consistent with sarcoma with BCOR genetic alterations and hence described as a Ewing-like sarcoma.

The patient had an uneventful recovery and was discharged home after 1 week of hospital stay. No adjuvant chemotherapy was required. She was scheduled for outpatient follow-up with the general surgeons and medical oncologist for ongoing review.

## Discussion

Gastric ES was first reported in the literature in 2004 [4]. At present, there are only 13 reported cases of gastric ES where seven cases were located in the gastric body, in which two of them were identified to be in the gastric lesser curvature [1, 3–14]. The rarity of gastric ES may be associated with its primitive messenchymal cell origin that has limited potential for neural differentiation [1, 13]. Its histogenesis remains unclear however the commonest molecular abnormality in gastric ES involves translocation of chromosomes 11 and 22, t(11;22) (q24; q12) [1]. This results in the fusion of ESWR1 gene and the Erythroblast Transformation Specific (ETS) proteins as transcription factors which regulate the target genes, leading to cell transformation and generation of tumors with ES morphology and gene expression [1, 14].

In the few reported cases, patients with gastric ES presented with abdominal pain, vomiting, and upper gastrointestinal hemorrhage with only one reported case of a palpable abdominal mass [2, 11]. CT imaging of the abdomen may demonstrate a large exophytic mass or a hypodense circumferential mass in the stomach [2]. Calcifications may be present in 25% of the extraskeletal ES cases [3].

Histopathology remains the gold standard of diagnosis for extraskeletal ES [1]. The histological features of ES include undifferentiated uniform small round cells, absence of nucleoli, fine granular chromatin pattern, and scant cytoplasm with variable degrees of neuroectodermal differentiation [1]. The diagnosis of extraskeletal ES depends on the presence of round cell morphology and positive immunohistochemistry findings such as CD99, FLI-1, vimentin, NKX2, ERG, neuron-specific enolase (NSE), Leu-7, and synaptophysin due to the lack of specific diagnostic marker [1]. Almost all ES cases have a positive immunohistochemical staining for monoclonal antibody to CD99 [11]. Our patient had positive immunohistochemistry staining for CD99, NKX2.2, Vimentin, and PAX8. The molecular abnormalities of chromosomes 11 and 22 should be confirmed via fluorescence in situ hybridization in all patients with possible diagnosis of extraskeletal ES [1].

There is no standard management protocol for extraskeletal ES at present [1]. Surgical resection and chemoradiotherapy with a chemotherapy regime of Vincristine, Doxorubicin, Cyclophosphamide, Ifosfamide, and Etoposide are the current management strategies for extraskeletal ES [1, 10, 13]. ES and extraskeletal ES are both radiosensitive [1]. Hence, post-operative radiotherapy may be considered for local disease control if there is difficulty in achieving clear tumor margins [1]. The 5-year event-free survival rate in patients who undergo both surgery and post-operative radiotherapy is also higher compared to patients who only had surgery (70% vs. 59%) [1].

In this report, we have highlighted the rare diagnosis of primary gastric Ewing-like Sarcoma. The current management of gastric ES is mainly guided by the histopathology results, radiological findings, and the extent of the disease. It is evident that the literature around primary gastric ES is scarce and thus this condition warrants further research so that management and diagnostic guidelines can be established.

## Conflict of interest statement

None declared.

## Funding

No funding sources.

## Data availability

All data analyzed during this study are included in this published article.
